# Draft Genome Sequences of Two Potentially Novel *Bacillus* Isolates from Backyard and Commercial Chicken Gastrointestinal Tracts

**DOI:** 10.1128/MRA.00492-20

**Published:** 2020-05-28

**Authors:** Jitendra Keshri, Rocio Ramirez, Mark E. Berrang, Brian B. Oakley

**Affiliations:** a College of Veterinary Medicine, Western University of Health Sciences, Pomona, California, USA; b Graduate College of Biomedical Sciences, Western University of Health Sciences, Pomona, California, USA; c USDA Agricultural Research Service, U.S. National Poultry Research Center, Athens, Georgia, USA; University of Rochester School of Medicine and Dentistry

## Abstract

Here, we present the draft genome sequences of two *Bacillus* strains, HF117_J1_D and USDA818B3_A, isolated in Pomona, California, from the gastrointestinal (GI) tract of backyard and commercial broiler chickens, respectively. The draft genomes of both strains appear to represent novel species.

## ANNOUNCEMENT

A total of 379 *Bacillus* species have been recognized as of 2019 (http://www.bacterio.net/bacillus.html). We isolated *Bacillus* sp. strain HF117_J1_D from fecal samples of a backyard chicken and strain USDA818B3_A from the cecal contents of a commercial broiler chicken in Pomona, California. Both strains were isolated aerobically at 37°C after 24 to 48 h of growth. Strain HF117_J1_D was grown on tryptic soy agar (TSA), and strain USDA818B3_A was grown on brain heart infusion (BHI) agar (BD, Franklin Lakes, NJ, USA). Genomic DNA was extracted with the TaKaRa NucleoSpin microbial DNA isolation kit. Isolates were initially identified as *Bacillus* spp. using 16S rRNA sequencing. The genomes of both strains were sequenced using the Illumina NovaSeq platform through the IIGB Genomics Core at the University of California, Riverside.

Dual-indexed libraries suitable for 150-bp paired-end reads on the NovaSeq platform were constructed using a plexWell 96 library preparation kit for Illumina sequencing platforms (seqWell, Inc., Beverly, MA, USA), and 1,667,450 and 4,753,763 paired-end 150-bp reads were generated for strains HF117_J1_D and USDA818B3_A, respectively. Default parameters for all software were used during analyses unless otherwise specified. Quality filtering of the reads was performed using Trimmomatic v0.39 (1). High-quality reads were used for *de novo* genome assembly with SPAdes v3.12.0 using the careful assembly method (2). Scaffolds were filtered for a minimum 200-bp read length and 30× coverage. The quality of the subsequent assemblies was assessed using QUAST (3). Genome completeness rates were found to be 92.7% and 99.38% for strains HF117_J1_D and USDA818B3_A, respectively, using CheckM lineage (4) and the Microbial Genomes Atlas (MiGA; http://microbial-genomes.org/) workflow (5). A search for similar genomes using the Mash/MinHash algorithm (6) showed the nearest reference representative genome to strains HF117_J1_D and USDA818B3_A to be Bacillus fordii DSM 16014 (GenBank accession no. ARGB00000000) (Mash distance, 16%) and Bacillus bataviensis 2482 (v2) (GenBank accession no. VIVN01000000) (Mash distance, 17%). The species identification tool SpecI (7), based on 40 universal single-copy marker genes, confirmed that both genomes could not be assigned to any species cluster. Taxonomic novelty at the species level for both genomes was verified against reference genomes of NCBI’s RefSeq database using MiGA analysis. The assembled genomes were annotated using the IMG/M Pipeline (8), and average coverage was calculated using the BBMap tool. Results of the assembly quality analysis conducted using the MiGA workflow based on 106 essential genes were excellent (85.1%) and high (78.2%) for HF117J1_D and USDA818B3_A, respectively. The assembly and annotation metrics are presented in Table 1.

**TABLE 1:** Assembly and annotation metrics

Statistic	Data for strain:	
	HF117_J1_D	USDA818B3_A
No. of quality-filtered paired-end reads	1,667,450	3,939,604
No. of scaffolds	210	90
*N*_50_ (bp)	48,679	270,360
Largest scaffold length (bp)	242,713	717,443
Assembly length (bp)	3,817,981	5,714,796
Avg genome coverage (×)	82	176
G+C content (%)	40.89	39.09
No. of rRNA genes	17	16
No. of tRNA genes	55	44
No. of functional protein coding genes	2,981	4,299
No. of hypothetical protein coding genes	808	1,208
No. of genes associated with KEGG pathways	1,068	1,505
No. of genes associated with KEGG orthology	1,829	2,705
GenBank accession no.	JAABNQ000000000	JAABNN000000000
SRA accession no.	SRR10903101	SRR10903100

Based on the “compare genome analysis” function in IMG, which uses genome-wide average nucleotide identity (ANI), the maximum pairwise ANI of strain HF117_J1_D with known genomes of *Bacillus* is 82.3% with Bacillus fordii DSM 16014 (GenBank accession no. ARGB00000000) (aligned fraction, 71.9%). The highest ANI of USDA818B3_A with known genomes of *Bacillus* is 83.1% with Bacillus bataviensis 2482 (v2) (GenBank accession no. VIVN01000000) (aligned fraction, 71.5%). The ANI between the two isolates is only 69.1%, suggesting that strains HF117_J1_D and USDA818B3_A are distantly related to each other. The availability of these genomes will be useful in further studies.

### Data availability.

The draft genome sequence and raw read sequences for both strains have been deposited in DDBJ/EMBL/GenBank under the accession numbers listed in Table 1.
